# High-throughput real-time PCR-based genotyping without DNA purification

**DOI:** 10.1186/1756-0500-5-573

**Published:** 2012-10-19

**Authors:** Anastasia Fedick, Jing Su, Chaim Jalas, Nathan R Treff

**Affiliations:** 1 Department of Molecular Genetics, Microbiology, and Immunology, UMDNJ-Robert Wood Johnson Medical School, Piscataway, NJ, USA; 2 Reproductive Medicine Associates of New Jersey, Morristown, NJ, USA; 3 Bonei Olam, Center for Rare Jewish Genetic Disorders, 1755 46th St, Brooklyn, NY, 11204, USA; 4 RMA of NJ ATTN, Anastasia Fedick 111 Madison Ave, Suite 100, Morristown, NJ, 07960, USA

**Keywords:** High-throughput, Genotyping, Blood, Sample-to-SNP, QuantStudio

## Abstract

**Background:**

While improvements in genotyping technology have allowed for increased throughput and reduced time and expense, protocols remain hindered by the slow upstream steps of isolating, purifying, and normalizing DNA. Various methods exist for genotyping samples directly through blood, without having to purify the DNA first. These procedures were designed to be used on smaller throughput systems, however, and have not yet been tested for use on current high-throughput real-time (q)PCR based genotyping platforms. In this paper, a method of quantitative qPCR-based genotyping on blood without DNA purification was developed using a high-throughput qPCR platform.

**Findings:**

The performances of either DNA purified from blood or the same blood samples without DNA purification were evaluated through qPCR-based genotyping. First, 60 different mutations prevalent in the Ashkenazi Jewish population were genotyped in 12 Ashkenazi Jewish individuals using the QuantStudio™12K Flex Real-Time PCR System. Genotyping directly from blood gave a call rate of 99.21%, and an accuracy of 100%, while the purified DNA gave a call rate of 92.49%, and an accuracy of 99.74%. Although no statistical difference was found for these parameters, an F test comparing the standard deviations of the wild type clusters for the two different methods indicated significantly less variation when genotyping directly from blood instead of after DNA purification. To further establish the ability to perform high-throughput qPCR based genotyping directly from blood, 96 individuals of Ashkenazi Jewish decent were genotyped for the same 60 mutations (5,760 genotypes in 5 hours) and resulted in a call rate of 98.38% and a diagnostic accuracy of 99.77%.

**Conclusion:**

This study shows that accurate qPCR-based high-throughput genotyping can be performed without DNA purification. The direct use of blood may further expedite the entire genotyping process, reduce costs, and avoid tracking errors which can occur during sample DNA purification.

## Findings

### Background

Many advances have been made in the field of genetics, including progress in high-throughput genotyping. The potential turnaround time due to the improved speed of genotyping however, is hindered by the slow upstream step of extracting genomic (g)DNA from blood samples. Manual or automated extraction of gDNA from blood can take at least several hours, without including the time it then takes to normalize each individual sample. Additionally, different volumes of blood are needed for different extraction methods, which are not always obtainable. Commercially available products from multiple vendors now allow for samples to be quickly prepared for genotyping without requiring DNA extraction. For instance, whole blood can be used directly in reactions, and has already been used for gel-based multiplex PCR experiments [1]. With this methodology, the need to obtain sample concentrations for normalization prior to use is eliminated. Additionally, as little as 1-10 uL of blood is required for the reaction, allowing for either less blood to be drawn or for a greater volume to be stored for future use. Products are also available for starting materials other than blood, and studies have been done directly on fungus to isolate DNA markers [2], and on viruses and pathogenic bacteria for PCR-based detection [3-5] without first purifying the DNA.

While these commercial methods have been validated for use with smaller throughput systems, such as gels or 96/384-well plates, they have not been tested on the recently developed high-throughput genotyping platforms. Therefore, this study investigates the performance of high throughput PCR-based genotyping directly from blood, without DNA purification. The results of this study will not only be informative for genotyping, but also for transitioning from low-throughput to high-throughput PCR-based experiments in other fields.

## Methods

In order to test the performance of high throughput PCR-based genotyping from blood, we incorporated a multiplex preamplification step of the targeted loci. Genotyping of >12,000 targets in one run was conducted through the use of the TaqMan Sample-to-SNP Kit and the QuantStudio 12K Real-Time PCR System (Life Technologies Inc. [LTI], Carlsbad, CA), which is representative of other available high-throughput PCR-based genotyping platforms. The blood and gDNA used in this study was obtained from individuals of Ashkenazi Jewish decent over the age of 18 with patient consent. Three 4 mL lavender tubes of blood were obtained for each patient through the butterfly phlebotomy method, and the blood samples were stored at 10-15°F until needed.

The 12 blood samples used in the initial validation had been collected, frozen, and stored over a period of time ranging from 1998-2009. For the purified samples, gDNA was extracted using the QIAamp DNA Blood Maxi Kit (QIAGEN Inc, Germantown, MD, USA) and the concentrations were obtained using a Nanodrop-8000 spectrophotometer (Thermo Fisher Scientific Inc., Wilmington, DE, USA). The samples were then normalized to 50 ng/uL. The unpurified samples were processed using the TaqMan Sample-to-SNP Kit protocol as recommended by the supplier (LTI). Two microliters of blood were used for each sample, and a primer pool was made consisting of 64 40X assays diluted 1:200. The 64 assays included 1 assay to detect a SNP on the Y-chromosome for gender identification purposes, 2 assays each for 3 different large deletion mutations [6], and 57 assays for 57 individual mutations. After pre-amplification, the product was diluted 1:20. Depending on the initial preparation method, two and a half microliters of sample was mixed with two and a half microliters of TaqMan OpenArray Genotyping Master Mix (for purified samples) or TaqMan GTXpress Master Mix (for unpurified samples) in a 384-well plate. The same samples were plated in duplicate for both methods. The samples were then loaded onto the OpenArray plate using the QuantStudio 12K Flex OpenArray AccuFill System (LTI). After real time (q)PCR and allelic discrimination, the results were analyzed using TaqMan Genotyper v1.2 software (LTI).

The results from both methods were analyzed separately due to method-based differences observed in clustering and signal intensities. Specifically, since the unpurified DNA underwent a preamplification step while the purified DNA did not, the unpurified DNA had higher signal intensities along both axes when compared to the purified DNA. The same samples were genotyped using both methodologies, however, so a direct analysis could be made between using normalized, purified DNA samples, or unpurified samples consisting of whole blood.

Of the twelve samples, five were known to be heterozygous carriers for one mutation each. The five mutations included a splicing mutation (1717-1G>T) [7] in the *CFTR* gene to cause Cystic Fibrosis, a nonsense mutation (Y231X) [8] in the *ASPA* gene to cause Canavan disease, a small deletion (delR608) [9] in the *SMPD1* gene to cause Niemann-Pick type A, and two frameshift mutations (84GG [10] and 1278insTATC [11]) in the *GBA* and *HEXA* genes to cause Gaucher Disease and Tay-Sachs, respectively. The remaining seven samples were known to be wild type for all of the mutations. The TaqMan assays used to genotype these mutations had been previously validated for use in our lab (unpublished observations). All of the assays were designed with the wild type allele having the VIC probe and the minor allele having the FAM probe.

A large-scale blinded study was then done on 96 blood samples in duplicate to further assess the validity of high-throughput genotyping directly from blood. The blood samples were processed using the TaqMan Sample-to-SNP Kit protocol as mentioned above and consisted of 58 male and 38 female samples collected, frozen, and stored over a period of time ranging from 1992-2011 (See Table 1). Two microliters of blood was used for each sample, and a primer pool was made consisting of the same 64 40X assays diluted 1:200 as in the initial study. After pre-amplification, the product was diluted 1:20. Two and a half microliters of the diluted product and two and a half microliters of TaqMan GTXpress Master Mix (LTI) were then mixed in a 384-well plate before being loaded onto the OpenArray plates in duplicate using the QuantStudio 12K Flex OpenArray AccuFill System (LTI). qPCR and allelic discrimination were then performed, and the results analyzed using TaqMan Genotyper v1.2 software.

**Table 1:** Collection dates and genders for the 96 blood samples

**Year Collected**	**Number of samples**	**Number of Males**	**Number of Females**
1992	1	1	0
1995	1	1	0
1996	2	1	1
1997	2	2	0
1999	1	0	1
2000	2	1	1
2001	1	1	0
2003	5	3	2
2004	1	1	0
2006	5	4	1
2007	5	3	2
2008	5	2	3
2009	8	5	3
2010	12	6	6
2011	45	27	18

Each of the 96 samples had prior genotyping results available through conventional genotyping methodologies. These 96 samples consisted of 95 new individuals, and one patient that had been included in the previous study. The same 60 mutations tested for in the initial study were genotyped in this study, which included missense, nonsense, frameshift, splicing, and small and large deletions.

## Results

**Figure 1:**
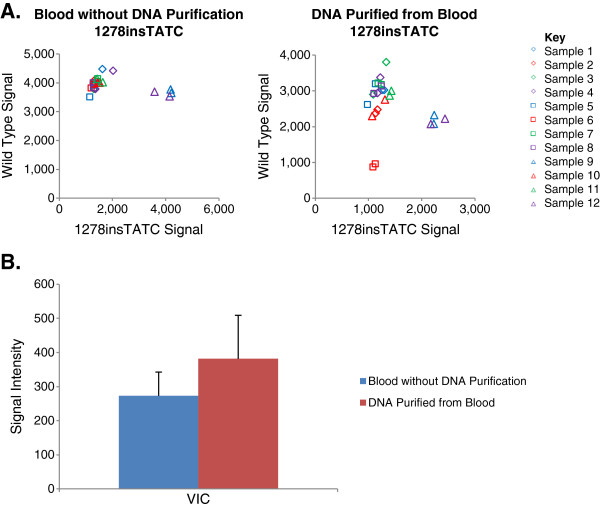
**Methodology Comparison.A**. Allelic discrimination plots depicting genotyping results for the same samples using either purified or unpurified DNA. The left panel shows genotyping directly from blood and the right panel shows genotyping after purification. The wild type allele used the VIC probe (y-axis) while the minor allele used the FAM probe (x-axis). **B**. The variance between samples was assessed by calculating the standard deviation of the wild type clusters for all of the assays. The results for genotyping directly are shown in blue and after purification are shown in red.

As indicated in both Figures 1A and Additional file 1: Figure S1, the results for the unpurified DNA samples analyzed using TaqMan Genotyper v1.2 software were equivalent in terms of cluster separation and correct genotype assignment to the results obtained for the same samples using purified DNA. Genotyping directly from blood gave a call rate of 99.21%, and both individual and replicate accuracies of 100%. Using purified DNA to genotype gave a call rate of 92.49%, an individual accuracy of 99.72%, and a replicate accuracy of 99.74%. The call rates were calculated by dividing the number of individual data points that had a genotyping call made by the total number of data points in the study. The lower call rate obtained when using the purified DNA stemmed largely from one sample that did not amplify for any of the assays when genotyped as purified DNA, even though it both amplified and provided accurate genotyping results when tested directly from blood. The individual accuracies were calculated by dividing the total number of individual data points with accurate genotype calls by the total number of genotype calls made, while the diagnostic accuracies consisted of the total number of replicates with both accurate and concordant genotyping calls divided by the total number of data points in the study. A two-tailed paired t-test was done to compare the call rates and accuracies of the two methods, which were found to be statistically insignificant at 0.391 and 0.182 respectively. This indicates that both methods detect and genotype a variety of polymorphisms equivalently, supporting the use of blood directly for PCR-based genotyping.

In order to compare the clustering between the same samples prepared using each methodology, the standard deviation was calculated for the wild type clusters of all 60 mutations (Figure 1B). Since the assays were designed with the VIC probe assigned to the wild type allele, the variance within the VIC signal was indicative of sample variance, while the signal levels of the minor allele probe (FAM) served as a negative control. As indicated by the bar graph, the samples genotyped directly from blood had less variation in the VIC signal when compared to the purified gDNA, signifying better clustering. The error bars consisted of the standard deviation of the standard deviations of the signals. A two-tailed F test indicated that the variation in the VIC signal was significant with a critical value of F equal to 7.24E-6. The variation in the FAM signal, however, was not significant with a critical value of F equal to 0.0596, which was expected since it served as the negative control.

**Figure 2:**
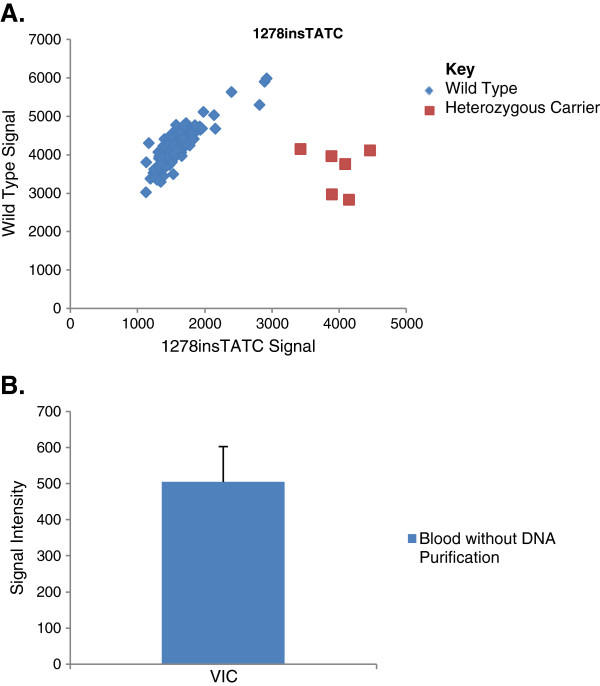
**Blind Validation Study.A**. An allelic discrimination plot depicting the genotyping results for 96 samples genotyped in duplicate. The wild type allele used the VIC probe (y-axis) while the minor allele used the FAM probe (x-axis). Figure 2**B**. Standard deviation of wild type clusters for the VIC signal. The variance between sample clusters was assessed by calculating the standard deviation of the wild type samples for all of the assays.

A large blind validation was then done to further investigate the potential for high-throughput PCR-based genotyping directly from blood. 96 samples were genotyped in duplicate for the same 60 mutations as tested for in the initial validation. Even with the increase in sample size, successful clustering was still observed for the samples (Figure 2A). An overall call rate of 98.38%, an individual data point accuracy of 99.50% and a diagnostic accuracy of 99.77% was observed when using blood directly to genotype. In order to assess concordance, the standard deviations of the wild type clusters for all 60 mutations were calculated (Figure 2B). The increased variation observed in the VIC signal when compared to the values generated by the 12 samples stems from the larger sample size.

## Discussion

The results of this study demonstrate that using blood directly to perform high-throughput PCR-based genotyping provides equivalent results to using purified, normalized gDNA. As indicated, the incorporation of the Sample-to-SNP procedure with genotyping on a PCR-based high-throughput platform yielded an accurate, automatable, process applicable to large scale genotyping operations. This particular genotyping system is capable of producing approximately 1.4 million genotypes in a single day. The main advantage to using this method includes avoiding purifying the DNA prior to genotyping, which is often the rate limiting step in otherwise high-throughput procedures. Other studies have been done on different high-throughput platforms using small amounts of DNA and whole genome amplification [12], but DNA extraction and purification was required. In this study, genotyping was done on whole blood without prior DNA purification. Additionally, the samples were preamplified with a primer pool consisting of the same TaqMan genotyping assays that were on the TaqMan OpenArrays. Because of this, only the specific target mutation sites were amplified to increase the target genetic material and the risk of allele dropout, which can occur during whole genome amplification [13], was eliminated.

Quality control measures for the large blind validation included gender identification by means of a SNP on the Y-chromosome. This assay, however, only resulted in the formation of two genotyping clusters based on the presence (amplification) or absence (non-amplification) of the SNP. Since samples do not always amplify during PCR-based reactions, a better method for gender identification would be to target the single base differences in the amelogenin genes as previously described [14]. This method allows two genotyping clusters to form based on the presence of the X and Y chromosomes, and also identifies unamplified samples. Since assays can easily be incorporated and removed from the array, we intend to replace our current gender detecting assay with this assay and investigate its potential as a more reliable gender determination technique. Other quality control measures in the experiment included running the samples in duplicate to help ensure that correct genotyping calls were assigned, with disconcordant replicate calls indicating that the sample needed to be rerun. The use of blood directly without purification also eliminated handling steps where sample identification could have been compromised, and reduced the risk of contamination.

An important aspect of this study included using blood samples that had previously been frozen and stored. It is the common practice of many laboratories to store frozen blood samples for later use. While these samples are still viable for extracting gDNA once thawed [15], we wanted to ensure that the freezing would not affect the genotyping procedure when using the blood directly. As the results indicated, using blood that had been previously frozen for up to twenty years did not negatively affect the genotyping results, further supporting such methods for general practice.

Using samples directly for high-throughput genotyping without DNA extraction will continue to be investigated by our lab. In the future, reference samples will be incorporated during the analysis to further aid in correctly genotyping samples, with the hope that eventually this methodology will be used to perform routine carrier screening in a truly high-throughput fashion. Potential benefits of using blood directly to genotype include saving time and money, while improving sample information tracking by avoiding purification steps where samples are transferred between multiple tubes. Since this study has shown that DNA purification is not necessary for accurate PCR-based genotyping, the direct use of blood can therefore be used to further expedite the entire genotyping process, especially when combined with a high-throughput genotyping platform.

## Availability of supporting data

The data sets supporting the results of this article are included within the article and its additional file.

## Abbreviations

gDNA: genomic DNA; qPCR: Real time PCR.

## Competing interests

The authors declare that they have no competing interests.

## Authors´ contributions

AF carried out the study and drafted the manuscript. JS carried out the study. CJ participated in the design of the study. NT conceived of the study, participated in its design and coordination, and helped to draft the manuscript. All authors read and approved the final manuscript.

## Supplementary Material

Additional file 1 **Figure S1.** Methodology Comparison. A. Allelic discrimination plots depicting genotyping results using purified DNA. B. Allelic discrimination plots depicting genotyping results using blood directly for the same samples genotyped in A. The wild type allele used the VIC probe (y-axis) while the minor allele used the FAM probe (x-axis). Click here for file

## References

[B1] Wagner FF Bittner R Petershofen EK Doescher A Müller TH Cost-efficient sequence-specific priming-polymerase chain reaction screening for blood donors with rare phenotypes Transfusion 2008 48 6 1169 1173 10.1111/j.1537-2995.2008.01682.x 18422854

[B2] Guo JR Schnieder F Abd-Elsalam KA Verreet JA Rapid and efficient extraction of genomic DNA from different phytopathogenic fungi using DNAzol reagent Biotechnol Lett 2005 27 1 3 6 10.1007/s10529-004-6294-x 15685411

[B3] Kramvis A Bukofzer S Kew MC Comparison of Hepatitis B Virus DNA Extractions from Serum by the QIAamp Blood Kit, GeneReleaser, and the Phenol-Chloroform Method J Clin Microbiol 1996 34 11 2731 2733 8897174 10.1128/jcm.34.11.2731-2733.1996 PMC229395

[B4] Li Y Mustapha A Evaluation of four template preparation methods for polymerase chain reaction-based detection of Salmonella in ground beef and chicken Lett Appl Microbiol 2002 35 6 508 512 10.1046/j.1472-765X.2002.01231.x 12460434

[B5] Rose HL Dewey CA Ely MS Willoughby SL Parsons TM Cox V Spencer PM Weller SA Comparison of Eight Methods for the Extraction of Bacillus atrophaeus Spore DNA from Eleven Common Interferents and a Common Swab PLoSOne 2011 6 7 e22668 10.1371/journal.pone.0022668 PMC3144239 21818364

[B6] Fedick A Su J Treff NR Development of TaqMan allelic discrimination based genotyping of large DNA deletions Genomics 2012 99 127 131 10.1016/j.ygeno.2012.01.003 22281206

[B7] Guillermit H Fanen P Ferec C A 3' splice site consensus sequence mutation in the cystic fibrosis gene Hum Genet 1990 85 450 453 10.1007/BF02428306 2210769

[B8] Kaul R Gao GP Aloya M Balamurugan K Petrosky A Michals K Matalon R Canavan Disease: Mutations among Jewish and Non-Jewish Patients Am J Hum Genet 1994 55 34 41 8023850 PMC1918221

[B9] Levran O Desnick RJ Schuchman EH Niemann-Pick type B disease. Identification of a single codon deletion in the acid sphingomyelinase gene and genotype/phenotype correlations in type A and B patients J Clin Invest 1991 88 3 806 810 10.1172/JCI115380 1885770 PMC295465

[B10] Beutler E Gelbart T Kuhl W Sorge J West C Identification of the second common Jewish Gaucher disease mutation makes possible population-based screening for the heterozygous state Proc Natl Acad Sci USA 1991 88 2 10544 10547 10.1073/pnas.88.23.10544 1961718 PMC52965

[B11] Myerowitz R Costigan FC The Major Defect in Ashkenazi Jews with Tay-Sachs Disease Is an Insertion in the Gene for the α-Chain of β-Hexosaminidase J Biol Chem 1988 263 35 18587 18589 2848800

[B12] Hollegaard MV Grove J Thorsen P Nørgaard-Pedersen B Hougaard DM High-Throughput Genotyping on Archived Dried Blood Spot Samples Genet Test Mol Bioma 2009 13 2 173 179 10.1089/gtmb.2008.0073 19371215

[B13] Treff NR Su J Tao X Northrop LE Scott RT Jr Single-cell whole-genome amplification technique impacts the accuracy of SNP microarray-based genotyping and copy number analyses Mol Hum Reprod 2011 17 335 343 10.1093/molehr/gaq103 21177337 PMC3097071

[B14] Tzvetkov MV Meineke I Sehrt D Vormfelde SV Brockmöller J Amelogenin-based sex identification as a strategy to control the identity of DNA samples in genetic association studies Pharmacogenomics 2010 11 3 449 457 10.2217/pgs.10.14 20235797

[B15] Mychaleckyj JC Farber EA Chmielewski J Artale J Light LS Bowden DW Hou X Marcovina SM Buffy coat specimens remain viable as a DNA source for highly multiplexed genome-wide genetic tests after long term storage J Transl Med 2011 9 91 10.1186/1479-5876-9-91 21663644 PMC3128059

