# Supplementary material for: High-throughput real-time PCR-based genotyping without DNA purification
Source: BMC Res Notes. 2012 Oct 19;5:573. doi: 10.1186/1756-0500-5-573 (PMC3505170; doi:10.1186/1756-0500-5-573)

Supplementary Figure 1. Methodology Comparison

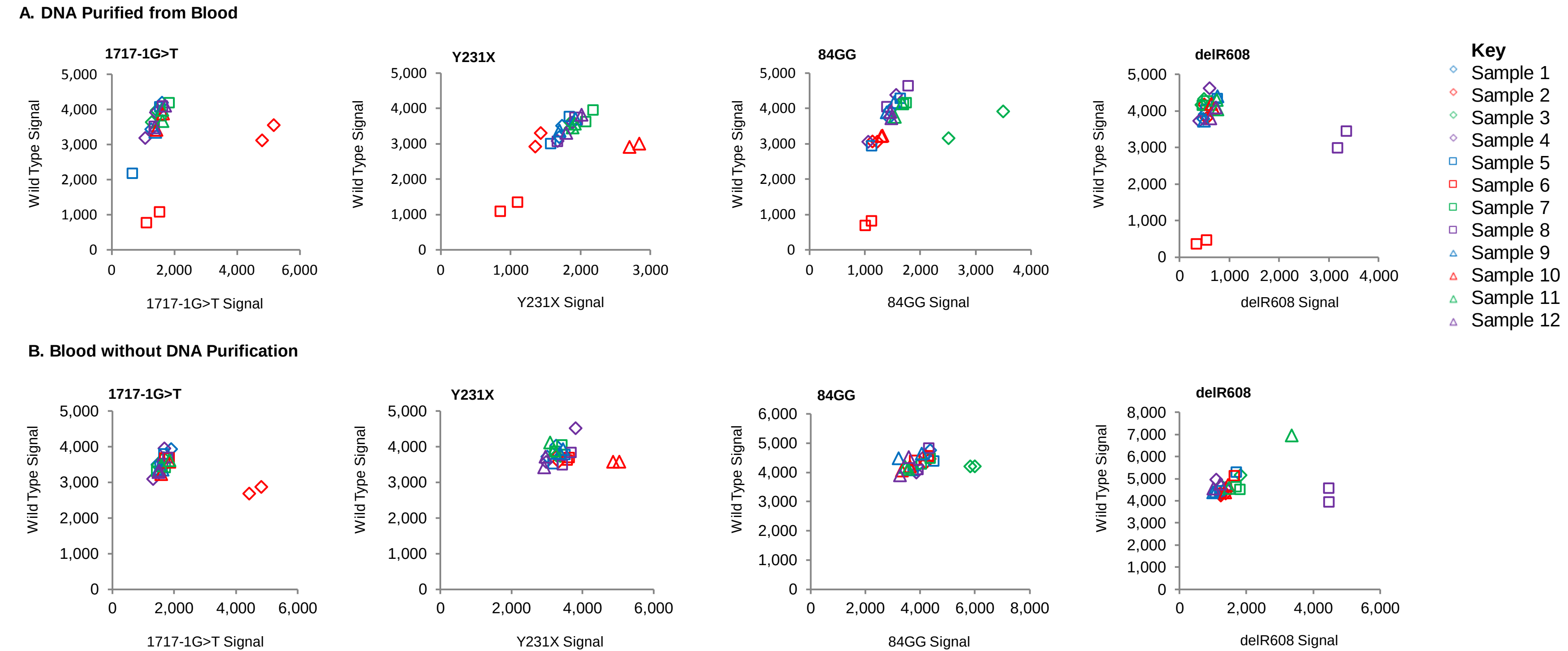

Supplement: Additional file 1 — Figure S1. Methodology Comparison. A. Allelic discrimination plots depicting genotyping results using purified DNA. B. Allelic discrimination plots depicting genotyping results using blood directly for the same samples genotyped in A. The wild type allele used the VIC probe (y-axis) while the minor allele used the FAM probe (x-axis). [file 1756-0500-5-573-S1.pdf]
